# Cerebellar involvement in Parkinson’s disease resting tremor

**DOI:** 10.1186/s40673-016-0051-5

**Published:** 2016-06-08

**Authors:** Shannon C. Lefaivre, Matt J. N. Brown, Quincy J. Almeida

**Affiliations:** Movement Disorders Research and Rehabilitation Centre, Wilfrid Laurier University, 75 University Avenue West, Waterloo, ON N2L 3C5 Canada; Department of Kinesiology, University of Waterloo, 200 University Avenue West, Waterloo, ON N2L 3G1 Canada

**Keywords:** Parkinson’s disease, Cerebellum, Tremor, rTMS

## Abstract

**Background:**

There exists a lack of consensus regarding how cerebellar over-activity might influence tremor in Parkinson’s disease (PD). Specifically, it is unclear whether resting or postural tremor are differentially affected by cerebellar dysfunction. It is important to note that previous studies have only evaluated the influence of inhibitory stimulation on the lateral cerebellum, and have not considered the medial cerebellum. The aim of the current study was to compare the effects of a low-frequency rTMS protocol applied to the medial versus lateral cerebellum to localize the effects of cerebellar over-activity.

**Methods:**

Fifty PD participants were randomly assigned to receive stimulation over the medial cerebellum (*n* = 20), lateral cerebellum (*n* = 20) or sham stimulation (*n* = 10). 900 pulses were delivered at 1Hz at 120 % resting motor threshold of the first dorsal interosseous muscle. Tremor was assessed quantitatively (before and after stimulation) using the Kinesia Homeview system which utilizes a wireless finger accelerometer to record tremor.

**Results:**

The main finding was that resting tremor severity was reduced in tremor-dominant individuals, regardless of whether stimulation was applied over the medial (*p* = 0.024) or lateral (*p* = 0.033) cerebellum, but not in the sham group.

**Conclusion:**

Given that the cerebellum is overactive in PD, the improvements in resting tremor following an inhibitory stimulation protocol suggest that over-activity in cerebellar nuclei may be involved in the generation of resting tremor in PD. Low-frequency rTMS over the medial or lateral cerebellum provides promise of an alternative treatment for tremor in PD, a symptom that is poorly responsive to dopaminergic replacement.

**Electronic supplementary material:**

The online version of this article (doi:10.1186/s40673-016-0051-5) contains supplementary material, which is available to authorized users.

## Background

Parkinson’s disease (PD) symptoms have generally been associated with dysfunction of the basal ganglia, and specifically, with the loss of dopaminergic producing neurons in the substantia nigra [1]. However, since increased activation levels of the cerebellum are also found in individuals with PD, it has been suggested that not all PD motor symptoms are due entirely to basal ganglia dysfunction [2–4]. Given the anatomical connections between the cerebellum and the basal ganglia, it has been suggested that increased cerebellar activity may also contribute to the pathophysiology of PD symptoms [2, 3, 5]. It is possible that increased excitatory output from the subthalamic nucleus in PD may be propagated to the cerebellum, via the pontine nuclei, and account for hyperactivity in cerebellar nuclei [3]. Understanding the effects of increased cerebellar nuclei activity in PD may be the key to gaining insight into some of the mechanisms underlying symptoms that are non-responsive or variably responsive to dopaminergic replacement.

Symptoms of bradykinesia and rigidity in PD demonstrate a stronger link with dysfunction of the basal ganglia and dopaminergic loss, while tremor seems to be less implicated with dopamine [2, 6, 7]. Given that tremor symptoms are generally less responsive to dopaminergic treatment, this might suggest that the pathophysiology of this symptom could be more related to over-activity in the cerebellum. Increased functional connectivity between the basal ganglia and cerebellum has been found in individuals with PD who experience tremor [8]; it has been suggested that while the basal ganglia may generate tremor, it is over-activity of the cerebellum that drives the tremor pattern [7]. In order to help understand the role of cerebellar over-activity in tremor, methods to suppress or reduce activity in the cerebellar nuclei provide a means to assess how PD symptoms might change under a temporary state of cerebellar depression.

The assessment of changes in tremor following repetitive transcranial magnetic stimulation (rTMS) protocols designed to transiently inhibit activity in the cerebellar nuclei are methods utilized in research to help determine whether it might be over-activity in the cerebellum contributing to this symptom. Other forms of TMS protocols have also been used to understand the involvement of the cerebellum in PD tremor. For example, tremor reset is a measure used to confirm the contribution of a brain area to either the generation or transmission of tremor. If the cortical target of TMS is involved in the pathophysiology of tremor, the effect of the stimulation interrupts the ongoing tremor drive and causes a tremor reset. A paired-pulse TMS protocol by Ni et al. (2010) demonstrated the involvement of the cerebellum in PD tremor by showing a reset of postural tremor following single-pulse cerebellar and primary motor cortex (M1) stimulation. It is important to note, however, that despite tremor reset, there was no change in tremor frequency following stimulation, and the effects were found only for postural, but not resting tremor after cerebellar stimulation [8]. In contrast, a study by Bologna et al. (2015) demonstrated no changes in resting tremor severity following continuous theta burst stimulation (cTBS) over the lateral cerebellum. This group suggested no involvement of the cerebello-thalamo-cortical loop in the generation of PD tremor [9]. Another study by Minks et al. (2011), which utilized one single session of low frequency (1Hz) rTMS over the right lateral cerebellum found significant improvement in bradykinesia of gross motor skills of both hands following stimulation. Although there was a benefit to gross motor skills, fine motor skills worsened and was only seen on the hand ipsilateral to stimulation [10]. A trial with greater clinical benefits using rTMS was that of Koch et al. (2009) which employed a two-week treatment of bilateral cerebellum using cTBS. This study resulted in reduced levodopa-induced dyskinesias (LIDs) for a period up to 4 weeks following the stimulation protocol [11]. Likewise, five consecutive days of transcranial direct current stimulation (tDCS) over the motor cortex and lateral cerebellum have also been shown to significantly reduce LIDs in individuals with PD [12]. These studies demonstrate a link between the modulation of cerebellar activity and some level of change in motor symptoms, suggesting the cerebellum may play a role in the pathophysiology of PD motor symptoms. The previous studies also demonstrate how inhibitory TMS protocols may be a useful tool for developing a better understanding of how the cerebellum might contribute to PD symptoms.

It is important to note that these previous studies applied stimulation targeted only to the lateral cerebellum, whereupon the cerebellar output nucleus (dentate) relays in the thalamus and basal ganglia. Additionally, these previous studies may not have had a pure sample of tremor dominant PD participants, which is important because not all individuals with PD present with tremor. Hence, this may have potentially lead to variable tremor results following cerebellar modulation. It may be beneficial to include a non-tremor dominant PD control group to ensure tremor results are definitive. Therefore, the aim of the current study was to use a low frequency rTMS protocol to transiently inhibit neural activity in the cerebellum of individuals with PD to further understand how over-activity in the cerebellum may contribute to tremor. Further, to localize the effects of over-activity in specific cerebellar nuclei, a direct comparison controlled by sham stimulation was done between the effects of stimuli applied over the medial versus lateral cerebellum. The assessment of changes in this less dopaminergic responsive PD motor symptom has the potential to uncover new knowledge about cerebellar pathophysiology in PD tremor. Further understanding of the involvement of cerebellar activity in PD has the potential to guide the development of treatments targeted to those symptoms which are less responsive to dopaminergic replacement therapy, such as tremor.

## Methods

### Participants

Fifty individuals diagnosed with idiopathic PD who met inclusion criterion were recruited for participation from the Movement Disorders Research & Rehabilitation Centre database (Wilfrid Laurier University, Waterloo, Ontario, Canada). For participant demographics, see Table 1. For safety, individuals who had received deep brain stimulation, implanted aneurysm clips or cochlear implants were excluded from participation. Previous history of seizures or the prescription of medications which lower the seizure threshold were also criterion for exclusion. Given the requirement to limit movements of the head and neck during stimulation, individuals with severe dyskinesia in the neck muscles were also excluded. Participants were blinded and randomized into three groups: Medial (*n* = 20), Lateral (*n* = 20) or Sham (*n* = 10). The Unified Parkinson’s Disease Rating Scale motor sub-section (UPDRS-III) scores for each participant were assessed by a blinded movement disorders specialist. This study was carried out in compliance with the Helsinki declaration and approved by the research ethics board at Wilfrid Laurier University (#4247), with all participants providing informed consent.

**Table 1:** Participant demographics; no significant differences

	Medial	Lateral	Sham
N	20 (4 F, 16 M)	20 (6 F, 14 M)	10 (3 F, 7 M)
Age (years)	69.4 (9.1)	66.8 (12.1)	71.1 (8.7)
UPDRS-III	22.7 (8.6)	23.1 (11.4)	19.5 (8.0)

### Stimulation protocol

Stimulation was delivered using a Magstim Rapid2 with 70 mm double air film coil (Magstim, UK) guided by TMS Manager navigation (Northern Digital Inc., Waterloo, Ontario). Stimulation intensity was based upon the resting motor threshold (RMT) of the right first dorsal interosseous (FDI) muscle. The measurement of resting electromyography (EMG) activity of the hand in individuals with PD is difficult due to the constant muscle activity from the generation of tremor, however, FDI has been shown in previous work to be capable of maintaining a level of muscle relaxation comparable to healthy individuals [8].

The motor hot spot for the FDI muscle was found in the primary motor cortex (M1) by systematically modifying coil placement and orientation until a consistently isolated FDI contraction was found. RMT was determined by decreasing stimulator output in 1 % intervals and defined as the lowest stimulus intensity which produced a motor evoked potential of 50–100 μV in amplitude in at least five out of ten consecutive responses with the hand relaxed [5, 13, 14]. RMT served to standardize the stimulus intensity relative to individual motor thresholds and varying levels of cortical excitability across participants.

The stimulation protocol consisted of one single session, where 900 pulses at 1Hz were applied at 120 % RMT. Previous studies have shown that 900 pulses is sufficient for cerebellar suppression [14, 15]. In the absence of individual magnetic resonance images to determine exact structure depth, the higher stimulation intensity was used to account for the increased distance between the coil and the cerebellum in comparison to the motor cortex [16, 17]. The stimulation target was either the cerebellar vermis (medial group) or lateral cerebellum (lateral group). The vermis is located directly beneath the inion, and was located through surface palpation. The lateral cerebellum, or dentate nucleus, is located three centimetres lateral and one centimetre inferior to the vermis [18]. Lateral cerebellar stimulation was applied to the side of the participant that was most affected by PD symptoms. Side affected was determined by self-report and confirmed by clinician assessment. All stimulation (both real and sham) was applied while participants were seated with their face resting on a padded surface, creating both a comfortable position for the participants, while at the same time minimizing head and neck movement. Sham stimulation was employed by angling the coil at 90° to the participants’ scalp, following the same protocol as real stimulation. All stimulation (real and sham) was delivered while participants were in their ON medication state.

### Outcome measures

Quantitative measures of resting and postural tremor were assessed for each hand using the Kinesia Homeview tablet, which is equipped with a wireless motion sensor that is placed on the index finger. The motion sensor consists of three accelerometers and three gyroscopes which are able to capture motion of the hands in the x,y and z planes at 128Hz. The output is then run through a previously validated algorithm by the Kinesia software and calculates a tremor severity score which ranges from 0 to 4 at a resolution of 0.1 [19–22]. This score, which has been demonstrated to be highly correlated with clinician ratings [21, 22], is based upon kinematic measures including peak power, frequency of peak power and root mean square of angular velocity. While in the seated position, tremor was recorded for 15 s separately for each hand. Participants were instructed to relax the hands between the legs to assess resting tremor, during which time the stroop task was presented to distract participants from tremor activity. Participants were instructed to raise both arms directly in front to shoulder height to assess postural tremor. Other outcome measures included the speed and amplitude of finger tapping, hand pronation/supination, index finger nose touch and the opening and closing of the fist. The starting position for all voluntary movements were standardized so that any movement were relative to this initial position. The movements were intended to mimic those performed during the upper limb portion of the UPDRS-III motor assessment.

### Analysis

Given that the presence of motor symptoms is heavily dependent on PD subtypes, a two-way repeated measures ANOVA with two independent factors was run to account for either the presence or absence of tremor in participants. This analysis compared stimulation (Lateral vs Medial vs Sham) and PD Subtype (Tremor Dominant (TD) vs Postural Instability Gait Impaired Dominant (PIGD)) and one within factor (Pre-assessment vs Post-assessment). This analysis was run for both hands combined, as well as the most affected hand by PD symptoms separately, to account for the ipsilateral effects expected from lateral stimulation of the cerebellum.

With the expectation that tremor symptoms would only improve in tremor-dominant participants, a second mixed repeated measures ANOVA comparing the effects of stimulation (Lateral vs Medial vs Sham) from pre-assessment to post-assessment using only TD patients (except for the Sham group) was run. Any significant findings were further examined with Tukey’s HSD post hoc procedure.

## Results

Tremor outcome data was not included from three participants, therefore statistical analysis was run on 19 participants who received medial stimulation, 20 participants who received lateral stimulation and eight participants who received sham stimulation (total 47 participants).

**Fig. 1:**
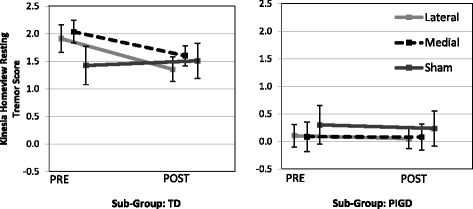
Resting tremor measures for both sides combined (*p* = .21770). Standard error of each measure represented by vertical bars. (TD: Tremor Dominant, PIGD: Postural Instability Gait Dominant)

**Fig. 2:**
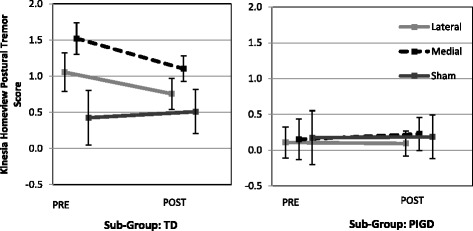
Postural tremor measures for both sides combined (*p* = .28207). Standard error of each measure represented by vertical bars. (TD: Tremor Dominant, PIGD: Postural Instability Gait Dominant)

**Fig. 3:**
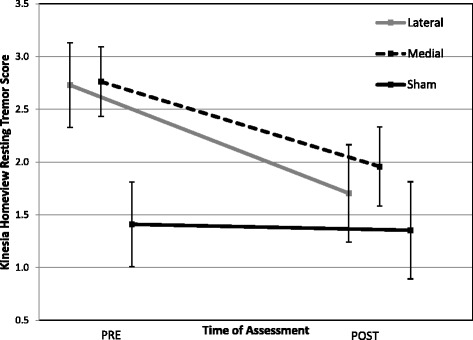
Resting tremor measures for the side most affected by Parkinson’s disease (PD) demonstrate near- significant group x time interaction (F(2,25) = 2.89, *p* = 0.074). Standard error of each measure represented by vertical bars

The two-way repeated measures ANOVA did not produce any statistically significant interactions, however the analysis did demonstrate a clear advantage for taking into account the presence of tremor as an independent factor. Graphical representation of the results of resting tremor showed no change in the PIGD group, meanwhile there appears to be a trend towards improvement in both rest and postural tremor in the TD participants regardless of whether the medial or lateral stimulation was applied. This result was consistent for analyses looking at the results from both hands combined (See Figs. 1 and 2), as well as for the hand most affected by PD tremor (See Fig. 3).

A mixed repeated measures ANOVA, including only TD participants, showed a near-significant time x stimulation interaction (F(2,25) = 2.89, *p* = 0.07), demonstrating a strong trend toward a decrease in tremor severity at post-assessment for both the medial and lateral stimulation groups (Fig. 3). Post hoc analysis revealed the medial stimulation group to have improved tremor by 29.1 % (*p* = 0.024) and lateral stimulation to have improved by 37.5 % (*p* = .033). Importantly, there was no change in the sham stimulation group, demonstrating this effect could not be attributed to placebo effects.

## Discussion

The aim of the current study was to use a low frequency rTMS protocol to transiently inhibit activity in the cerebellar nuclei of individuals with PD to gain an understanding of how localized over-activity in the cerebellum may contribute to tremor symptoms. The effects of stimulation were assessed based upon changes in tremor symptoms, where a change or improvement following inhibitory stimulation might suggest the involvement of cerebellar over-activity in the generation of tremor.

To date, this is the first cerebellar rTMS protocol to demonstrate improvements specifically in resting tremor in individuals with PD. Our results show that the severity of resting tremor was reduced in individuals regardless of whether stimulation was applied over the medial or lateral cerebellum (although it appears that the lateral cerebellum was the more effective target). This effect was specific to individuals with PD who were tremor-dominant, suggesting involvement of the cerebellum in the generation of resting tremor. This is in contrast to previous work which utilized continuous theta burst stimulation and found no change to resting tremor frequency, concluding the cerebello-thalamo-cortical circuit to have no involvement at all in the generation of resting tremor [9]. Another study which utilized a paired-pulse stimulation paradigm, suggested the isolated involvement of the cerebellum in postural tremor only, and not resting tremor [8]. This might suggest the generation of resting and postural tremor to be from different pathways. Findings from the current study, however, provide evidence which supports the theory that hyperactivity in the cerebellum contributes to resting tremor in PD. Key differences in the current study such as an objective measure of tremor, the comparison between medial and lateral stimulation, and analysis by tremor-dominance, may have enabled these findings.

Given that a low frequency, inhibitory stimulation was applied, improvements in resting tremor may be attributed to the potential normalization of the activation level in the cerebellar circuitry. The cerebello-thalamo-cortical pathway might allow transmission of cerebellar excitability changes to the thalamus, basal ganglia and M1. Thus, there are two pathways in which the cerebellum may interrupt the ongoing tremor drive: i) cerebellum-thalamus-primary motor cortex, or ii) cerebellum-thalamus-basal ganglia loop-primary motor cortex. Interestingly, inhibitory stimulation applied over the medial or lateral cerebellum both benefit resting tremor by supressing the output from either the dentate or fastigial nucleus in PD (though results suggest lateral stimulation to be slightly more effective). Knowing that tremor has previously been shown to be unrelated to levels of striatal dopamine depletion [7], this study supports the theory that the pathophysiology of tremor-dominant PD may be less associated with dysfunction of the basal ganglia and more related to over-activity in the cerebello-thalamo-cortical circuit [6].

A few limitations exist within the outcome measure chosen to assess tremor. It would have been beneficial to have the inability to extract kinematic parameters from the motion sensory individually for analysis as opposed to the output of only a composite score. Understanding whether the improvement in resting tremor was driven mainly by a decrease in tremor frequency or a change in tremor amplitude is important from a clinical perspective. Irrespective of decreasing tremor frequency, a change in the amplitude of the tremor movements might enable greater functional control of the hands during activities of daily living. The Kinesia motion sensor has however been proven to have a strong correlation with clinical tremor ratings, in addition to having the granularity to detect changes in tremor severity that were not distinguishable by clinician assessment [22].

To build upon the current evidence, an important extension of this study would be to recruit a larger sample, consisting of only tremor-dominant PD participants. This would provide adequate power to assess exactly how significant the improvement might be in this PD sub-group. Further, it is important to understand what other effects the stimulation may have had on the PIGD sub-group. Since there was no improvements in tremor expected in the PIGD sub-group, an assessment of how the stimulation may effect gait and balance symptoms should be considered. The assessment of changes in gait and balance outcome measures would also be important for the TD sub-group, since the cerebellum is an important subcortical structure implicated in the control of gait and balance. Understanding of the more global effect this stimulation may have on less-dopaminergic responsive PD symptoms, encompassing tremor, gait and balance would help to determine the therapeutic potential of low frequency inhibitory stimulation.

## Conclusion

Overall, this study suggests the involvement of the medial and lateral cerebellum in the generation of resting tremor in PD. These single sessions of inhibitory stimulation over either the medial or lateral cerebellum provide evidence to suggest that long-term application of the inhibitory protocol, consisting of multiple rTMS sessions, could potentially produce longer lasting benefits. Understanding the mechanisms underlying the cerebellar pathophysiology in PD has the potential for developing new treatments for symptoms which are less responsive to dopaminergic replacement.

## Abbreviations

μV, Microvolts; ANOVA, analysis of variance; cTBS, continuous theta burst stimulation; EMG, electromyography; FDI, first dorsal interosseous; Hz, Hertz; M1, Primary Motor Cortex; PD, Parkinson’s disease; PIGD, postural instability gait dominant; RMT, resting motor threshold; rTMS, repetitive transcranial magnetic stimulation; TD, tremor dominant; UPDRS, unified Parkinson’s disease rating scale; LID, levodopa-induced dyskinesia

## Additional file

Additional file 1: Summary Analysis Data. (DOCX 13 kb)
