# Supplementary material for: Cerebellar involvement in Parkinson’s disease resting tremor
Source: Cerebellum Ataxias. 2016 Jun 8;3:13. doi: 10.1186/s40673-016-0051-5 (PMC4897799; doi:10.1186/s40673-016-0051-5)
Supplement: Additional file 1: — Summary Analysis Data. (DOCX 13 kb) [file 40673_2016_51_MOESM1_ESM.docx]

**Summary Analysis Data:**

TD: Tremor Dominant; PIGD: Postural Instability Gait Dominant

|  | | **Medial, TD** | **Medial, PIGD** | **Lateral, TD** | **Lateral, PIGD** | **Sham** |
| --- | --- | --- | --- | --- | --- | --- |
| **Resting Tremor** | | | | | | |
| *Both Hands Combined* | Pre | 2.035134271 (0.91655) | 0.085525934 (0.09755) | 1.910335867 (1.03924) | 0.104412899 (0.13626) | 0.862348 (0.67716) |
|  | Post | 1.600774942 (0.65271) | 0.081590107 (0.0713) | 1.352798004 (1.0698) | 0.051866872 (0.08723) | 0.871508 (0.80928) |
| **Resting Tremor** | | | | | | |
| *Most Affected Side* | Pre | 2.761598831 (0.99276) | 0.111298346 (0.13325) | 2.729170753 (0.98907) | 0.052460903 (0.07235) | 1.409991 (1.25906) |
|  | Post | 1.957202072 (1.15299) | 0.120490182 (0.124561) | 1.704181181 (1.10917) | 0.048589201 (0.05936) | 1.352643 (1.44173) |
| **Postural Tremor** | | | | | | |
| *Both Hands Combined* | Pre | 1.521304443 (1.18881) | 0.150244898 (0.027508) | 1.05501703 (0.70085) | 0.108075721 (0.09057) | 0.300192 (0.47965) |
|  | Post | 1.102166037 (0.91581) | 0.227791656 (0.277104) | 0.755372464 (0.6365) | 0.093129237 (0.0921) | 0.348406 (0.46771) |
| **Postural Tremor** | | | | | | |
| *Most Affected Side* | Pre | 1.826978855 (1.35956) | 0.223390583 (0.4.1912) | 1.392821034 (0.84025) | 0.13760128 (0.17249) | 0.460927 (0.77381) |
|  | Post | 1.509136722 (1.24567) | 0.284214175 (0.310765) | 1.03509288 (0.90203) | 0.097575275 (0.10002) | 0.586966 (0.86946) |
